# Clinical characteristics and evolution of 71 neonates born to mothers with COVID-19 at a tertiary center in Brazil

**DOI:** 10.1016/j.clinsp.2022.100136

**Published:** 2022-11-02

**Authors:** Bruna de Paula Duarte, Vera Lucia Jornada Krebs, Valdenise Martins Laurindo Tuma Calil, Werther Brunow de Carvalho, Maria Augusta Bento Cicaroni Gibelli, Rossana Pulcineli Vieira Francisco

**Affiliations:** a Departamento de Pediatria da Faculdade de Medicina da Universidade de São Paulo, São Paulo, SP, Brazil; b Departamento de Ginecologia e Obstetrícia Faculdade de Medicina da Universidade de São Paulo, São Paulo, SP, Brazil

**Keywords:** Neonatology, COVID-19, Newborn, Brazil

## Abstract

**Introduction:**

Limited data are available on pregnant women with COVID-19 and their neonates.

**Objective:**

This study aimed to describe clinical characteristics and evolution from birth to discharge of a retrospective cohort of 71 neonates, with one set of twins, born to women with COVID-19 diagnosed at the end of pregnancy. The authors included all newborns admitted into a neonatal unit of a tertiary hospital in Brazil, between March 2020 and March 2021, whose unvaccinated mothers had COVID-19 symptoms and RT-PCR (Real-Time Polymerase Chain Reaction) for SARS-CoV-2 positive within fourteen days prior to delivery. Newborns to mothers with COVID-19 symptoms and negative tests for SARS-CoV-2 were excluded.

**Results:**

The main route of birth delivery was cesarean, corresponding to 60 pregnant women (84.5%). The foremost indications for cesarean were pregnant with critical disease (24.6%) and acute fetal distress (20.3%). The mean birth weight was 2452 g (865‒3870 g) and the mean gestational age was 34^5/7^ weeks (25‒40 weeks). There were 45 premature newborns (63.3%), of which 21 newborns (29.5%) were less than 32 weeks of gestational age. RT-PCR for SARS-CoV-2 on oropharyngeal swabs was positive in 2 newborns (2.8%) and negative in the other 69 newborns (97.2%). Most newborns (51.4%) needed respiratory support. Therapeutic interventions during hospitalization were inotropic drugs (9.9%), antibiotics (22.8%), parenteral nutrition (26.8%), and phototherapy (46.5%).

**Conclusion:**

Maternal COVID-19 diagnosticated close to delivery has an impact on the first days of neonatal life.

## Highlights

• This study's main objective is to describe the clinical characteristics and evolution from birth to discharge of 71 neonates born to unvaccinated women with COVID-19 with RT-PCR for SARS-CoV-2 positive within fourteen days prior to delivery in a Brazilian hospital.
• The neonatal SARS-CoV-2 positivity rate was 2.8% and these newborns had few clinical symptoms.
• The prematurity rate was 63.4%, indicating that maternal disease may lead to neonatal complications associated with a higher prematurity rate.

## Introduction

In December 2019, a disease caused by the new coronavirus or SARS-CoV-2 was described: as COVID-19,^1^^,^^2^ recognized as a pandemic by the World Health Organization (WHO) in March 2020.^3^ At this time, large medical centers prioritized research with the general population. Therefore, studies focused on pregnant women and their newborns are inconclusive, since they were mainly based on case series.^4^, ^5^, ^6^

Viral infections during pregnancy can be risk factors for maternal and fetal complications.^7^, ^8^, ^9^ However, several publications about SARS-CoV-2 have not shown a worse clinical evolution in these populations^6^^,^^10^, ^11^, ^12^ and there is no robust evidence of vertical transmission of SARS-CoV-2 in late pregnancy.^13^, ^14^, ^15^, ^16^ Nevertheless, identification of SARS-CoV-2 in the placenta, fluids, and neonatal secretions has been described,^16^, ^17^, ^18^, ^19^ as well as low rates of test positivity are reported in newborns, who commonly present with nonspecific clinical symptoms, radiological and laboratory findings. Most newborns to mothers with COVID-19 have a negative test result for SARS-CoV-2 and usually have a benign course.^20^ Larger studies are still needed to better elucidate neonatal disease findings.

The aim of the study was to describe the clinical findings at the beginning of life of newborns born to women diagnosed with COVID-19 at the end of pregnancy and to analyze the possible association between the severity of maternal disease and the neonatal clinical outcome.

## Methods

The authors conducted a retrospective cohort analysis of all 71 neonates, with one set of twins, born to 70 symptomatic unvaccinated mothers with positive RT-PCR (Real-Time Polymerase Chain Reaction) for SARS-CoV-2. The test was collected by nasopharyngeal swab between 14 days prepartum until the delivery. All newborns were admitted to the Neonatal Center of Children's Institute of Clinical Hospital of Medicine's University of São Paulo between March 2020 and March 2021. Neonates born to symptomatic women with a negative RT-PCR test for SARS-CoV-2 were excluded. All data were collected from electronic medical records. This study was approved by the ethics committee of the hospital involved in the study (Certificate of Presentation of Ethical Appreciation: 43592021.2.0000.0068).

The gestational age was based on the date of the last period or calculated by first-trimester ultrasound. Timing and way of delivery were determined by an obstetrician in terms of obstetric indications (vaginal, cesarean or forceps). The authors reported the critical women's disease at the delivery: respiratory failure with invasive respiratory support and/or shock requiring inotropes.

The newborn characteristics were described, such as weight, head circumference, and length at birth and Fenton growth charts classification. Each newborn was classified at birth as small for gestational age (<10th percentile), appropriate for gestational age (10th to 90th percentile), or large for gestational age (>90th percentile). Also, Apgar test, the need for neonatal resuscitation requiring positive pressure ventilation, orotracheal intubation, cardiac compressions, umbilical catheterization, and/or use of vasoactive agents in the delivery room.

Additionally, the authors considered supportive treatments such as oxygen therapy, advanced respiratory support (non-invasive/invasive), use of inotropic drugs, parenteral nutrition, phototherapy, and antibiotic indication in the first 72 h of life.

The newborn's fluid samples were taken by a staff who was trained and designated by the NICU (Neonatal Intensive Care Unit). Staff performing invasive procedures (airway aspiration, intubation, respiratory sample) used disposable waterproof gowns, N95 masks, goggles/eye protection, and gloves. Hand hygiene was attained before and after gloves. Pharyngeal samples were tested for SARS-CoV-2 with the RT-PCR method following WHO guidelines, 48 h after birth and at subsequent times when necessary.

Patients were discharged by NICU criteria based on the recommendations of the Brazilian Society of Pediatrics.^21^^,^^22^

### Statistical analyses

The comparison between qualitative variables was performed using the Chi-Square test or Fisher's exact test. When applicable, the nonparametric Mann-Whitney test was used. Odds Ratios (OR) and their respective 95% Confidence Intervals (95% CI) were estimated. A value of *p* < 0.05 was considered significant. Data were analyzed using SPSS version 23 for Microsoft Windows.

## Results

From March 2020 to March 2021, 71 neonates born to symptomatic mothers and a positive test for SARS-CoV-2 were admitted at the tertiary neonatal center.

The main perinatal and neonatal characteristics are described in Tables 1 and 2.

**Table 1:** Perinatal and neonatal characteristics of 71 neonates born to mothers with COVID-19.

Perinatal and neonatal characteristics		n (%)
Maternal		
Type of delivery	Cesarean	60 (84.5)
	Vaginal	10 (14.1)
	Forceps	1 (1.4)
Ceserean indication	Mild, moderate or severe disease^a^	8 (11.6)
	Critical disease^a^ with mechanical ventilation or inotropes required	17 (24.6)
	COVID and fetal distress	14 (20.3)
	COVID and oligohydramnios	6 (8.7)
	Others	13 (18.8)
Mechanical ventilation	Yes	20 (28.6)
Antenatal corticosteroid	Yes	12 (16.9)
Neonatal		
Gender	Female	38 (53.5)
	Male	33 (46.5)
Neonatal resuscitation	Yes	22 (31.4)
Respiratory support	No	34 (48.6)
	Supplemental oxygen	3 (4.3)
	CPAP^b^	16 (22.9)
	Mechanical ventilation	17 (24.3)
Inotropes	Yes	7 (9.9)
Antibiotics < 72 h of life	Yes	20 (28.2)
Parenteral nutrition	Yes	19 (26.8)
Phototherapy	Yes	33 (46.5)
Neonate RT-PCR^c^ SARS-CoV-2	Positive result	2 (2.8)
Neonatal mortality	‒	2 (2.8)

a WHO classification^23^.

b Continuous Positive Airway Pressure*.*

c Real-Time Polymerase Chain Reaction.

**Table 2:** Distribution of quantitative clinical variables at birth of 71 neonates born to mother with COVID-19.

Neonatal variables	Mean	SD^a^	Minimum	Median	Maximum
Lenght (centimeters)	44.3	4.7	30.0	45.8	52.0
Length percentile^b^	34.5	24.3	1.0	29.0	81.0
Head circumference (centimeters)	32.1	3.2	21.0	33.0	37.0
Head circumference percentile^b^	59.5	28.4	1.0	60.0	99.0
Weight (grams)	2452	864	580	2500	3870
Weight percentile^b^	50.0	24.6	2.0	55.0	97.0
Apgar at 1 min	‒	‒	0.0	8.0	10.0
Apgar at 5 min	‒	‒	1.0	9.0	10.0

a Standart Deviation.

b Fenton growth charts classification.^24^

**Fig. 1:**
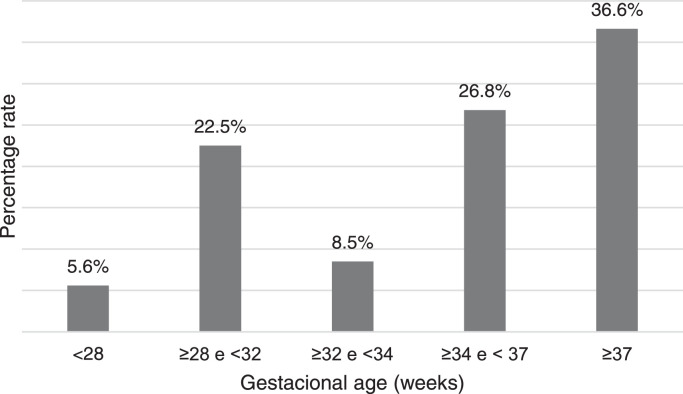
Distribution of quantitative clinical variables of 71 newborns to mothers with COVID-19.

The prematurity rate was 63.3% (45 newborns) and its distribution according to gestational age is shown in Fig. 1. The mean gestational age at birth was 34 weeks and 5 days and the median was 35 weeks.

According to Fenton's growth charts (24) classification, there were 3 newborns (4.2%) small for gestational age, 67 (94.3%) appropriate for gestational age, and 1 (1.4%) large for gestational age. Regarding birth weight, there were 4 newborns (5.6%) with extremely low weight, 6 (8.4%) with very low weight, and 25 (35.0%) with low weight.

There were 2 deaths (2.8% mortality rate), both RT-PCR negative for SARS-CoV-2 collected between 24 and 48 h of life.

The first one was a female newborn, delivered vaginally, with gestational age at birth of 25 weeks and 1 day, whose mother had mild symptoms of COVID-19 at the time of delivery. The neonate's birth weight was 580 g, had a 1st and 5th minute Apgar scores of 1 and 7, respectively, and was intubated in the delivery room. The transthoracic echocardiogram showed an atrial septal defect without hemodynamic repercussions and the cranial ultrasound showed grade II intracranial hemorrhage. She died at 37 days of life due to late neonatal sepsis. The other newborn was male, delivered by C-section indicated by maternal hemodynamic instability, and was on mechanical ventilation at the time of delivery. Gestational age at birth was 28 weeks and 1 day and birth weight were 700 g. The 1st and 5th minute Apgar scores were 1 and 8, respectively, and he was intubated in the delivery room. The transthoracic echocardiogram showed a patent foramen ovale. The death occurred at 13 days of life due to late neonatal sepsis.

Cesarean was the way of delivery in 84.5% of the cases. There was no significant difference between the delivery route and the neonatal variables analyzed.

In Table 3, critical disease and fetal distress were the variables that stood out in terms of chance of events, namely: neonatal resuscitation, Apgar test, and respiratory support.

**Table 3:** Association between delivery indication and perinatal and neonatal characteristics (*n* = 71).

Perinatal and neonatal characteristics	Delivery indication	OR^a^ (95% CI^b^)	p
Neonatal resuscitation at birth	Mild, moderate or severe disease^c^	1.0	
	Critical disease^c^ with mechanical ventilation or inotropes required	1.8 (0.14‒23.83)	0.643
	COVID and fetal distress	15.7 (2.76‒89.55)	***0.002***
	COVID and oligohydramnios	14.6 (2.44‒88.13)	***0.003***
	Others	2.2 (0.17‒29.31)	0.551
Apgar at 1 min ≤ 7	Mild. moderate or severe disease	1.0	
	Critical disease with mechanical ventilation or inotropes required	1.0 (0.09‒11.24)	1.000
	COVID and fetal distress	10.0 (2.13‒47.02)	***0.004***
	COVID and oligohydramnios	5.2 (1.05‒26.20)	***0.043***
	Others	1.4 (0.12‒16.46)	0.789
Apgar at 5 min ≤ 7	Mild. moderate or severe disease	1.0	
	Critical disease with mechanical ventilation or inotropes required		
	COVID and fetal distress	16.1 (1.74‒148.67)	***0.014***
	COVID and oligohydramnios	9.2 (0.91‒93.02)	0.060
	Others		
Respiratory support	Mild. moderate or severe disease	1.0	
	Critical disease with mechanical ventilation or inotropes required	0.8 (0.13‒5.03)	0.821
	COVID and fetal distress	7.2 (1.74‒30.55)	***0.007***
	COVID and oligohydramnios	6.0 (1.42‒26.03)	***0.015***
	Others	12.1 (1.19‒123.62)	***0.035***
Parenteral nutrition	Mild. moderate or severe disease	1.0	
	Critical disease with mechanical ventilation or inotropes required		
	COVID and fetal distress	2.7 (0.63‒11.78)	0.179
	COVID and oligohydramnios	6.6 (1.48‒30.11)	***0.014***
	Others	1.0 (0.09‒11.03)	1.000

a Odds Ratio

b Confidence Intervals 95%.

c WHO classification.^23^

There was a statistically significant association between maternal mechanical ventilation and the need for neonatal resuscitation (Table 4). Odds ratio analysis shows that, when the mother needed mechanical ventilation, the chance of neonatal resuscitation was 103.5 (95% CI 17.4–615.4; *p* < 0.001), and the chance of Apgar test at the 5th minute < 7 was 36 (95% CI 8.6–150.9). In this analysis, preterm newborns were excluded to minimize sampling bias.

**Table 4:** Association between maternal invasive mechanical ventilation and neonatal characteristics.

Perinatal and neonatal characteristics	Maternal mechanical ventilation (n = 20)		
	n (%)	p	OR (95% IC)
Neonatal resuscitation at birth	18 (90.0)	***< 0.001***	103.5 (17.4‒615.4)
Apgar at 1 min ≤7	16 (80.0)	***< 0.001***	26.8 (6.6‒108.8)
Apgar at 5 min ≤7	12 (60.0)		36.0 (8.6‒150.9)
RT-PCR SARS-CoV-2 test	0 (0.0)	1.000	

*Premature were excluded.

The length of hospital stays of the newborns ranged from 2 to 194 days (mean: 20.1 days; median: 7.5 days), with a significant association between the length of stay and each of the therapeutic interventions.

The neonatal positivity rate of the RT-PCR test for SARS-CoV-2 collected from oropharyngeal swabs between 24 and 48 h of life was 2.8% (2 newborns). All tests were confirmed with a second sample. The clinical findings in two neonates with positive RT-PCR tests for SARS-CoV-2 are described in Table 5.

**Table 5:** Clinical findings in two neonates with positive RT-PCR test for SARS-CoV-2.

Clinical findings	Case 1	Case 2
Gender	Male	Female
Gestational Age (weeks)	38 ^4/7^	33 ^4/7^
Maternal disease classification^a^	Mild	Severe
Maternal mechanical ventilation	No	No
Maternal death	No	No
Route of delivery	Vaginal	Cesarean
Neonatal resuscitation at birth	No	No
Weight / Classification^b^	2980 g/AGA^3^	2130 g/AGA^3^
Apgar at 1/5 min	9/9	9/9
Respiratory support	No	Oxygen therapy
Signs. symptoms and labs	Bradycardia e hypocalcemia	Hypoxemia e enterorrhagia
Echocardiograma	Patent foramen ovale	Patent foramen ovale
Positive RT-PCR SARS-CoV2	2nd e 3rd day of life	2, 3 e 17th day of life
Leght of hospital stay	8 days	26 days

a WHO classification.^23^

b Fenton growth charts classification.^24^ ^c^Appropriate for Gestational Age.

Among the 35 newborns who underwent cranial ultrasound, the following results were observed: no abnormal findings (71.0%), grade I or II periventricular hemorrhage (22.0%), ventricular dilatation (2.8%), and linear calcifications (2.8%). The findings in the transthoracic echocardiogram, performed in 36 newborns, were: patent foramen ovale (69.4%), atrial septal defect (27.7%), and ventricular septal defect (2.7%).

## Discussion

Among the 71 newborns born to mothers with a confirmed COVID-19, the RT-PCR test positivity rate for SARS-CoV-2 was 2.8%. This result is lower than that observed by Papapanou et al.^25^, in a multicenter cohort study whose neonatal positivity rate was 13%, with an association between cesarean delivery and a positive test. Other authors also reported higher transmission rates than those observed in the present study. Chi et al.^13^, in a systematic review of 230 pregnant women with COVID-19, among whom there were 154 deliveries, observed a vertical transmission rate of SARS-CoV-2 of 3.91%. In this study, in addition to the oropharyngeal swab, serological tests were performed to search for neonatal antibodies against SARS-CoV-2.

To date, there is no diagnostic standardization to establish vertical transmission of SARS-CoV-2. Schwartz et al.^26^ propose that the confirmation of the transplacental passage can be achieved by the virus identification in the chorionic villi, through immunohistochemistry. Vivanti et al.^17^, described the detection of SARS-CoV-2 in the placenta of pregnant women with COVID-19 through RT-PCR. A meta-analysis^6^ evaluated 16 observational studies and 44 case reports, suggesting that the rate of vertical transmission is low, unrelated to the severity of maternal illness or delivery route. The RT-PCR positivity rate for SARS-CoV-2 obtained by oropharyngeal swabs among newborns of mothers with COVID-19 ranges from 1.6% to 10%.^10^^,^^17^^,^^25^^,^ ^27^

Preterm births have been associated with SARS-CoV-2 positive tests in pregnant women^5^^,^^20^^,^^28^ and neonatal complications in this population are related to the high rate of prematurity. In a cohort^29^ of neonates born to mothers with COVID-19 at the time of delivery, a significant association between positive maternal SARS-CoV-2 test and increased risk of neonatal diseases was demonstrated. Some authors^25^ highlighted the significant increase in morbidity in newborns born to women with COVID-19 compared to children born to mothers without the disease. In the present study, the mean gestational age at birth was 34 weeks and 5 days and the gestational age was less than 32 weeks in 29.5% of the newborns. The prematurity rate was 63.4%, higher than the 9.9% rate reported in Brazil in 2019.^30^ This was the main finding in the present study, indicating that maternal COVID-19 at the time of delivery can have serious clinical consequences for the newborn, even with a negative neonatal result of RT-PCR for SARS-CoV-2.

The authors had two newborns with a positive test result for SARS-CoV-2.^31^ The clinical findings were nonspecific with few symptoms, as described by other authors.^5^^,^^17^^,^^25^^,^^27^ However, there are reports of neonates with an unfavorable evolution.^32^

The cesarean section rate (84.5%) was considered high if compared to WHO rates^33^ that predate the COVID-19 pandemic. A systematic review carried out after 2019 describes a cesarean section rate of 52.3% to 95.8%,^34^ although in several studies there is no clear description of the indication for cesarean delivery.

The main indication for cesarean delivery was a maternal critical disease with respiratory and/or hemodynamic failure in 24.6% of pregnant women. Twenty pregnant women (28.6%) required mechanical ventilation at delivery and maternal intubation was associated with a higher rate of need for neonatal resuscitation.

In comparison with most studies, the rate of Low-Birthweight (LBW) infants observed in the present series (49%) was higher than that described by other authors.^5^^,^^25^^,^^35^ Conversely, a systematic review^34^ describes similar findings. The high proportion of LBW newborns in the present series was in agreement with the prematurity rate.

Data on the indication for neonatal resuscitation reported by other authors are divergent.^5^^,^^10^^,^^36^ The results obtained in the present study show that 31.4% of newborn required neonatal resuscitation at birth. Metz et al.^28^ demonstrated that pregnant women with severe COVID-19 were at greater risk of adverse perinatal outcomes, such as prematurity and NICU admission. Among 255 neonates in this study, those whose delivery was indicated due to maternal COVID-19 had worse outcomes, including a higher rate of prematurity and a greater need for ventilatory support.

This study has certain limitations. The present study involved a single center in an underdeveloped country. Furthermore, the present cohort had a reasonably small number of participants.

## Conclusion

The prematurity rate of 63.4% of newborns to unvaccinated mothers with COVID-19, indicates that maternal disease may lead to neonatal complications associated with a high rate of prematurity. The neonatal SARS-CoV-2 positivity rate was low (2.8%), and the newborns had few clinical symptoms.

## Conflicts of interest

The authors declare no conflicts of interest.
